# New Resin-Based Bulk-Fill Composites: *in vitro* Evaluation of Micro-Hardness and Depth of Cure as Infection Risk Indexes

**DOI:** 10.3390/ma13061308

**Published:** 2020-03-13

**Authors:** Marco Colombo, Simone Gallo, Claudio Poggio, Vittorio Ricaldone, Carla Renata Arciola, Andrea Scribante

**Affiliations:** 1 Department of Clinical, Surgical, Diagnostic and Paediatric Sciences – Section of Dentistry, University of Pavia, Piazzale Golgi 2, 27100 Pavia, Italy; marco.colombo@unipv.it (M.C.); vittorio.ricaldone01@universitadipavia.it (V.R.); andrea.scribante@unipv.it (A.S.); 2 IRCCS Istituto Ortopedico Rizzoli, Laboratorio di Patologia delle Infezioni Associate all’Impianto, via di Barbiano 1/10, 40136 Bologna, Italy; 3 Department of Experimental, Diagnostic and Specially Medicine, University of Bologna, via San Giacomo 14, 40126 Bologna, Italy

**Keywords:** bulk-fill composites, micro-hardness, hardness ratio, depth of cure, acid exposure, bacterial colonization, infection

## Abstract

The current *in vitro* study evaluated the Vickers hardness number (VHN) and hardness ratio of four bulk-fill composites (VisCalor bulk; Admira Fusion x-tra; x-tra fil; and GrandioSO x-tra-Voco, Cuxhaven, Germany) to assess the risk of bacterial colonization in comparison with standard composite materials. Thirty samples were prepared for each group. The VHN of both the external (top) and internal surface (bottom) was determined with a micro-hardness tester (200 g load for 15 s), and the hardness ratio was also calculated for each sample. Subsequently, storage in an acidic soft drink (Coca-Cola, Coca-Cola Company, Milano, Italy) was performed; for each group, 10 samples were stored for 1 day, while another 10 were stored for 7 days and the remaining 10 were kept in water as controls. A significant reduction in VHN was shown for all the groups when comparing the external versus internal side (P < 0.05), although the hardness ratio was greater than 0.80, resulting in an adequate polymerization. Regarding the acid storage, all the groups showed a significant decrease of VHN when compared with the controls, both after 1 day (P < 0.05) and after 7 days (P < 0.001). All the products showed adequate depth of cure without further risk of bacterial colonization. However, acid exposure negatively affected micro-hardness values, which might promote subsequent colonization.

## 1. Introduction

Resin-based composites (RBCs) are nowadays the most used materials for the direct restoration of teeth, mainly due to their aesthetic properties, which are extremely appreciated when compared to those of other materials historically used in dentistry [1]. Traditionally, RBCs require an incremental apposition of 2 mm-layers, both to allow the curing light to penetrate the material and to contrast the shrinkage that takes place during polymerization [2]. Because of the multiple stratifications necessary, this technique may be quite time-consuming, especially when big cavities of the tooth must be filled. Therefore, in recent years, manufacturers have tried to develop new products requiring single-layer stratification, thus reducing the operative steps. Resin-based “bulk-fill” composites (BFCs) have been introduced with this purpose, and according to manufacturers, can be used in a single step to fill cavities of a 4 mm depth or greater [3]. However, when choosing composite resins, clinicians should consider the risks of secondary decay and cytotoxicity that are still associated with them [4,5].

The first objection to BFCs concerns their effective depth of cure, considering the gradual decrease in the degree of conversion from the external surface exposed to the curing light to the internal one [6], with scattering and absorption being the major factors related to light attenuation [7]. A low depth of cure has been showed to compromise the color stability, but above all, the mechanical and biological properties of the restorations [7,8]. As with the mechanical ones, in fact, a degree of conversion below a minimum threshold of 55% is considered insufficient, thus causing less resistance to wear [9]. Instead, regarding the biological properties, an incomplete curing of the composite might be responsible for bacterial colonization. In fact, the release of monomers (such as EGDMA and TEDGMA) from incompletely cured composites has been reported to promote the proliferation of cariogenic bacteria [10], as well as being associated with the cytotoxicity of gingival and pulp fibroblast cells [11]. Considering that residual monomers can be released even in well-polymerized RBCs [12], a major amount might elute from BFCs.

In the present *in vitro* study, we aimed to indirectly evaluate the depth of cure of four bulk-fill composites, assessing the Vickers hardness number (VHN) and hardness ratio. These parameters have been considered, since a significant correlation between increasing hardness and depth of cure is generally regarded [8,13]. Subsequently, the change in the surface micro-hardness of the tested materials following two alternative acid storages of 1 and 7 days respectively, was compared with the control samples that were stored in water.

The first null hypothesis of this study is that there is no significant difference in the hardness ratio of the new materials if compared with well-polymerized traditional RBCs, thus achieving a value of at least 0.80, as considered in literature [12,14]. Furthermore, the second null hypothesis is that no significant difference in micro-hardness values occurs after the two respective acid storages, compared to the controls.

## 2. Materials and Methods

Four composite resins (Voco, Cuxhaven, Germany) were selected for the present study: VisCalor Bulk (Group 1); Admira Fusion x-tra (Group 2); x-tra fil (Group 3); and GrandioSO x-tra (Group 4). The specifications of the materials and their acronym codes are listed in Table 1. For each product, the A2 Vita shade was chosen to reduce the effects of colorants on light-curing.

Thirty samples for each group were prepared by inserting the respective BFC into a stainless-steel mold (Ø 7 mm, h 4 mm) placed on a dark opaque paper background, with a polyester matrix strip interposed. This arrangement was chosen to obtain a smooth surface under the composite, but also to avoid light reflection from the bottom, thus minimizing the artificial hardening of this area [15]. Considering its termoviscous behaviour, VisCalor Bulk was preheated before the application with the preheating device (Caps Warmer, Voco, Cuxhaven, Germany) at 68 °C for 15 min, in accordance with the operating instructions.

Each mold was slightly overfilled, and a second polyester matrix strip (Mylar strip, Henry Schein, Melville, NY, USA) was placed on the top to prevent oxygen from interfering with the polymerization of the most superficial layer of the composite [16]; in order to extrude the excess composite resin and obtain a flat surface, a glass slide was pressed against the upper polyester film, and removed before curing [17].

Each sample was light-cured with the LED unit Celalux 2 (Voco, Cuxhaven, Germany), and was removed from the mold without undergoing polishing. Before every use, the cordless curing unit was maintained at full charge, and irradiance was checked with a radiometer (SDS Kerr, Orange, CA, USA). The distal end of the light guide was placed perpendicular to the surface of the matrix strip, and positioned concentrically with the mold, before starting the light-curing of the samples, which only took place on their external (top) side [18]. Exclusively one light polymerization mode was used (standard mode), with an output irradiance of 1000 mW/cm^2^ for 20 s, which are considered the minimum parameters to polymerize 4 mm increments of bulk-fill composites [19].

In order to simulate the physiological oral conditions, the samples were stored for 48 h in complete darkness, at 37 °C and 100% humidity, before proceeding with the assessment of the Vickers hardness number (VHN) [14]. This period has been awaited, as the polymerization of composite resins continues at a slow rate even after curing [8].

After removing the Mylar strip, the VHN of the upper and lower surface of each sample was determined with a micro-hardness tester (durometer Isoscan HV2, LTF Spa, Antegnate, Bergamo, Italy) equipped with a Vickers diamond indenter to apply a 200 g load with a 15 s dwell time [20]. Three indentations were equally placed over a circle, with each being no closer than 0.5 mm to the adjacent indentation (UNI EN ISO 6507) [21]. A 40× magnification built-in scale microscope was used to measure the two diagonals of each indentation. The VHN was calculated in kgp/mm^2^ using the following equation: HV = 1.854 P/d2, where P is the load in kgf and d is the average length in mm of the diagonals. For a given specimen, a single hardness value was reported for each of the two surfaces, resulting from the mean of the three respective hardness values assessed. Furthermore, the hardness ratio of the specimens was calculated using the formula: hardness ratio = mean VHN of bottom surface / mean VHN of top surface.

**Figure 1:**
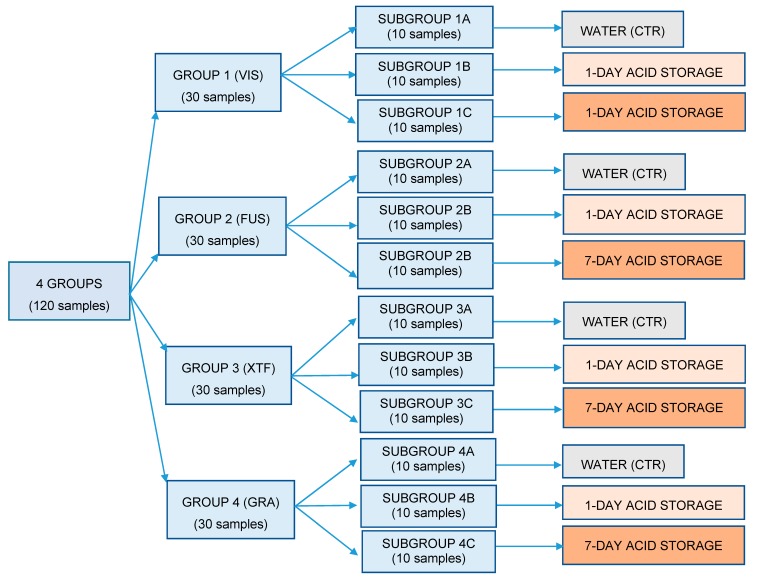
Flow chart showing how samples were divided into groups and subgroups and the respective treatment assigned. CTR: subgroup of control.

After the procedure described, the thirty samples of each group were divided into three subgroups (n = 10 per subgroup), as showed in Figure 1: each first subgroup (1A–4A) was kept in water during this experimental phase and was considered as control, while the other two subgroups underwent an acid storage in a soft drink (Coca-Cola, Coca-Cola Company, Milano, Italy) conserved at room temperature (18 ± 1 °C). In particular, the former (subgroups 1B–4B) were left inside for 1 day, while the latter (subgroups 1C–4C) were left for 7 days, with a daily renew of the soft drink.

At the end of the acid storage, the subgroups underwent hardness testing performed as previously described, but in this case, considering only the upper surface of the samples, which represents the portion of the material directly exposed to an erosive action in the oral cavity.

Data were submitted to statistical analysis using computer software (R version 3.1.3, R Development Core Team, R Foundation for Statistical Computing, Wien, Austria). Descriptive statistics, including the mean, standard error of mean, median, minimum and maximum values, were calculated for all groups. The normality of distributions was assessed with a Kolmogorov–Smirnov test. A multi-factor analysis of variance (ANOVA) and Tukey tests were applied to show differences among groups. Significance for all statistical tests was predetermined at P < 0.05. A linear regression model for Vickers hardness was performed, adding as a covariate the immersion time in the acidic drink.

## 3. Results

Descriptive statistics of the tested materials’ micro-hardness after light curing are shown in Table 2. The acronym codes are reported as given in Table 1. 

**Figure 2:**
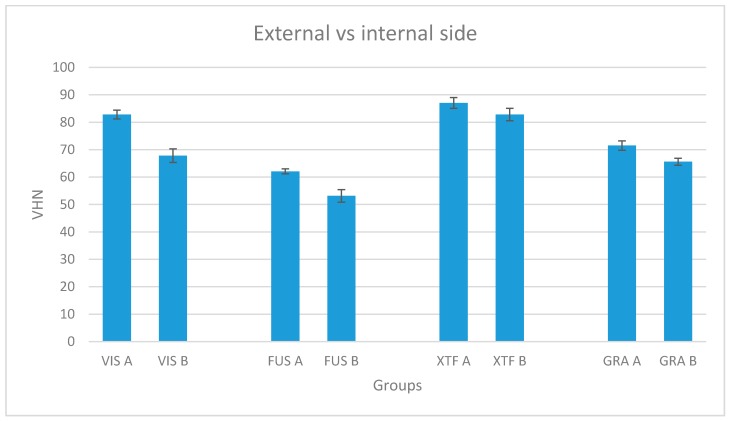
Mean Vickers hardness number and standard deviation for each tested material, considering both the external and the internal side of the samples. A: External side; B: Internal side.

Considering the four bulk-fill composites (VIS, FUS, XTF and GRA) and comparing the external (top, A) versus the internal side (bottom, B) of the samples, a significant difference in the mean percentage reduction of VHN was shown for all the respective groups tested (P < 0.05) (Figure 2), with 18.14% for group 1 (VIS), 14.40% for group 2 (FUS), 4.79% for group 3 (XTF) and 8.22% for group 4 (GRA) (Table 3), in accordance with their hardness ratio (0.82, 0.86, 0.95 and 0.92, respectively), as shown in Table 4. Despite this reduction between the values of the top and bottom sides, the hardness ratio was higher than 0.80 for all the groups.

**Figure 3:**
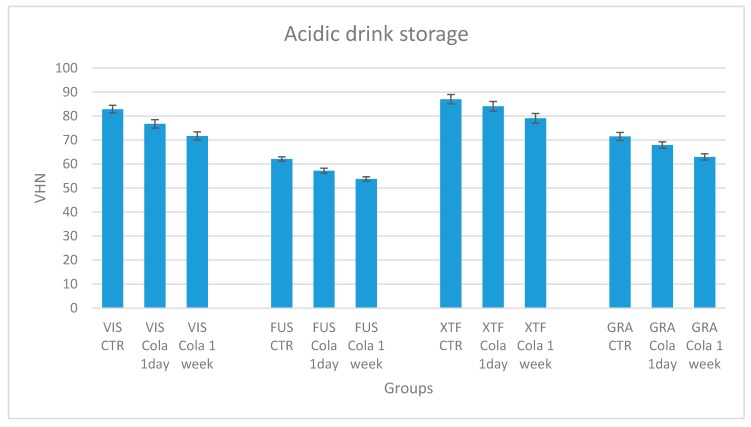
Mean Vickers hardness number and standard deviation for each tested material, considering the two different acid storages compared to a subgroup of control.

Descriptive statistics of the micro-hardness after acid storage are shown in Table 5. When evaluating the acidic drink storage, all the tested products showed a significant decrease of VHN, both after the 1-day storage (P < 0.05) and the 7-day storage (P < 0.001) in Coca-Cola, compared to the control subgroups stored in water (Figure 3). Meanwhile, solely for group 1 (VIS) and group 2 (FUS), the mean percentage micro-hardness losses after the first day of immersion (T0–T1), which were 7.30% and 7.83% respectively, were higher than those which took place during the following six days (T1–T2), which were 6.58% and 5.97% respectively, although these latter values are not significantly different if compared to the former. On the contrary, in accordance with other studies assessing a major micro-hardness loss of restorative dental materials during the first seven days of acid exposure [22], an opposite trend has been reported for groups 3 and 4 (Table 6)—even in this case, however, no significant difference was shown comparing the micro-hardness losses at the two times considered. Anyway, the mean percentage reduction of VHN at the end of the 1-day acid storage only appeared to be significantly lower for group 3 if compared with the other groups at the same time, while it was significantly lower for both groups 3 and 4 considering the 7-day acid storage.

The linear regression model (Table 7) shows that the Vickers hardness was significantly affected by the immersion time in an acidic drink (P < 0.0001).

## 4. Discussion

Since the introduction of light-cured resin composites, several studies have showed that their degree of light-curing depends on both intrinsic and extrinsic factors, with the former including the photoinitiator system, optical properties, filler, type and amount of monomer, while the latter includes the light spectrum, light irradiance, irradiation time and mode, temperature and light guide tip positioning [23]. Adequate polymerization is considered a crucial factor for the success of the restoration, since an insufficient curing degree is responsible for water absorption, decreasing wear resistance and strength, and the elution of uncured monomer with a toxic effect [24,25].

Studies assessing the depth of cure are essential, even in the lower surfaces of the materials, as the cure of composite restorations can only be evaluated by clinicians in the upper surface that are directly exposed to the curing light [26,27]. Both direct and indirect methods have been proposed to evaluate the depth of cure. Among the latter, micro-hardness correlates well with the degree of conversion of resin composites [28,29], although an absolute hardness number cannot be used to directly compare different resins [14]. Conversely, other methods such as optical microscopy and scraping [30] have been shown to overestimate the depth of cure [6,31].

Traditional resin-based composites (RBCs) are used in dentistry with a 2 mm-incremental technique, which has been shown to be effective in ensuring well-polymerized layers [32]. However, many defects have been discussed, such as the failure of bonding between the layers, the presence of free spaces and a possible contamination among them [8]. Anyway, one of the most relevant disadvantages related to this technique is the difficulty of restoring small cavities, as well as the lengthy application time [33]. In order to avoid these factors, bulk-fill resin composites have been proposed in recent years with the potentiality of being cured with thickness of 4 mm or even more, according to manufacturers. These materials are characterized by particular properties which allow the polymerization of layers of such a depth.

One of the purposes of this study was to verify the effective depth of cure of four bulk-fill resin composites, in order to estimate a possible risk of bacterial colonization and secondary decay associated with the uncured monomer elution from these materials; in fact, an inverse correlation between the depth of cure and monomer elution occurs [8].

The micro-hardness value of the external side of the tested materials was significantly different between the groups, and was reported, in increasing order, for Admira Fusion x-tra, GrandioSO x-tra, VisCalor Bulk and x-tra fil. Considering the internal side, the values were reported in the same order, but with no significant difference between VisCalor Bulk and GrandioSO x-tra.

Since the only VHN cannot be considered as an index of the depth of cure, this was assessed in this study through the hardness ratio. All groups have showed a value greater than 0.80, which is the minimum to consider traditional RBCs as adequately cured [12,14].

As regards VisCalor Bulk and Admira Fusion x-tra, the hardness ratios were 0.82 and 0.86, respectively. This shows that, despite the latter material being characterized by lower micro-hardness numbers, the values of its two opposite surfaces are more similar between them than if compared to those of VisCalor Bulk, resulting in a hardness ratio nearer to 1. This can be explained considering the intrinsic factors of the material, such as a combination of the conventional photoinitiator system with one that is more sensitive [16], as well as a higher translucency, allowing a major amount of curing light to penetrate and polymerize the material homogeneously [34]. In particular, the reduction of the refractive index difference existing between the base resin and filler is related to an improvement of the degree of conversion and depth of cure [35,36].

Incidentally, the highest hardness ratios reported in this study were for GrandioSO x-tra and x-tra fil, respectively, with 0.92 and 0.95. For the same reasons previously reported for Admira Fusion x-tra, GrandioSO x-tra shows a similar hardness ratio to x-tra fil, despite its lower micro-hardness values. 

According to these results, the first null hypothesis of this study has been accepted, since all the tested bulk-fill composites—particularly x-tra fil and GrandioSO x-tra—guarantee an adequate degree of conversion when polymerized in layers of 4 mm, despite only a polymerization mode being used in the present study.

We can assert that these materials do not present a greater risk compared with traditional RBCs, with regards to bacterial colonization and the subsequent risk of secondary infection of the tooth. Moreover, in accordance with this, other studies reported that despite a major amount of cuspal deflection [37], bulk-fill composites seem to show no negative influence on marginal integrity [38].

Considering that a low pH due to acidic drinks and foods influences the material’s mechanical and chemical properties [39], the second purpose of the study was to verify the micro-hardness change of the external side of the materials after two different acid storages, of 1 day and 7 days, respectively, carried out in Coca-Cola (pH = 2.52) at room temperature (18 ± 1 °C). The exposure to the acid drink was continuous, in order to simulate long-term exposure to carbonated beverages in the oral cavity. In fact, immersion in Coca-Cola for only one day is analogous to its consumption for a month, considering the time it remains in the mouth while drinking [40].

In both storages, the micro-hardness of all tested materials was negatively affected. After both the 1-day and the 7-day acid storage, one of the highest mean percentage losses in micro-hardness was reported for VisCalor Bulk (of 7.30% and 13.42%, respectively). Despite its greater mean percentage reductions, however, this material reported a final micro-hardness value even higher than GrandioSO x-tra not exposed to the acidic drink, which is due to the higher initial micro-hardness value of the former material. 

Instead, regarding Admira Fusion x-tra, even though the mean percentage reductions were similar to those of VisCalor Bulk, its micro-hardness values were the lowest after the two acid storages among all the tested products, due to its low initial microhardness value. Finally, x-tra fil was the bulk-fill composite which showed the highest micro-hardness value before being exposed to the acid, and considering it also had the lowest mean percentage reductions after both acid storages, it is the material least affected by the acidic drink in the present study.

In conclusion, the second null hypothesis of this study has been rejected. As reported by other authors, the degradation of the composites’ polymer network, as well as the falling out of the filler due to the continuous action of acidic beverages, are responsible for the composites’ loss of micro-hardness [41,42,43,44]. Despite Coca-Cola being the soft drink most commonly used to study the properties’ change of restorative materials, other substances like food stimulating solvents [45] should also be tested.

Based on the importance of enamel and bone hardness, since bacteria takes advantage of micro-alterations [46], a decrease of the material’s hardness following the erosive action of acids might also be responsible for bacterial colonization. The biofilm accumulated over the restoration produces acidic substances, which further cause a surface degradation with the subsequent softening and roughening of the material’s surface [46,47]. Finally, a greater penetration of microorganisms through the fissure interposed between the restoration and the enamel may occur, resulting in the re-infection of the tooth (secondary caries) [48,49,50]. However, other factors influence this process under *in vivo* conditions, such as the buffering capacity of saliva, which decreases the erosive action of acids, as well as having a remineralizing effect on the tooth [51].

Moreover, the potential benefit of a better understanding of tooth decay by acidic drinks and/or acidifying bacteria that colonize and erode the teeth could extend to the knowledge of the pathogenesis of bone diseases in both the dental/periodontal and orthopedic fields. Indeed, bacteria-induced acidosis and metabolic acidosis can impinge on bone healing, negatively affecting the osteoblast-osteoclast equilibrium, collagen synthesis and bone matrix mineralization. Bone remodeling is not only controlled by cells, but also by the extracellular *milieu*, where calcium phosphate salts precipitate in a pH-dependent manner. Bone infection acidifies the *milieu* and promotes the formation/activation of osteoclasts, thus allowing hypothesizing that local pH changes may play a significant role in the pathogenesis of bone resorption and severely destructive osteomyelitis.

The limitations of the current report are related to the fact that the present study was completely conducted *in vitro*; in fact, laboratory experiments cannot duplicate intraoral conditions. Additionally, only bulk fill composites have been tested in the current report, and no traditional materials have been considered. Finally, a single acidic solution has been tested.

Further prospective *in vivo* studies should be performed to confirm the efficacy of bulk-fill resin composites in avoiding a major risk of secondary decay, related to the release of uncured monomers, but also to the loss of micro-hardness following exposure to acid.

## 5. Conclusions

Despite the limitations of this *in vitro* study, all the tested resin-based bulk-fill composites have shown an adequate depth of cure, which does not prefigure a major risk of secondary decay in comparison to traditional composites. As also reported for the latter, micro-hardness values appeared significantly decreased after both 1-day and 7-day acid storage, which may facilitate the subsequent bacterial colonization of the restorations, with a risk of secondary decay.

## Figures and Tables

**Table 1:** Characteristics of the materials tested in this study.

Group	Material	Code	Type	Composition	Filler Content %	Lot #	Manufacturer
1	VisCalor bulk	VIS	Termoviscous bulk-fill composite (nanofilled composite)	*Matrix*: Bis-GMA, aliphatic dimethacrylate *Filler*: Inorganic filler	83 (w/w)	76292	Voco, Cuxhaven, Germany
2	Admira Fusion x-tra	FUS	Nano-hybrid ORMOCER®-based material	*Matrix*: ORMOCER® *Filler*: glass ceramics, silica nanoparticles, pigments	84 (w/w)	1750435 1904427	Voco, Cuxhaven, Germany
3	x-tra fil	XTF	Light-curing posterior filling material	*Matrix*: dimethacrylate (Bis-GMA, TEGDMA, UDMA) *Filler*: Inorganic filler (Bariumaluminium silicate, fumed silica, pigments)	86 (w/w)	1906144	Voco, Cuxhaven, Germany
4	GrandioSO x-tra	GRA	Aestethic nanohybrid bulk restorative material	*Matrix*: Bis-GMA, Bis-EMA, aliphatic dimethacrylate *Filler*: Inorganic filler, organically modified silica	86 (w/w)	1907626	Voco, Cuxhaven, Germany

**Table 2:** Descriptive statistics of VHN for each tested material, considering both the external and the internal side of the samples.

Group	Material Code	Side	Mean	Min	Mdn	Max
1	VIS	External (A)	82.82 (1.62) ^a^	80.10	83.21	85.10
1	VIS	Internal (B)	67.80 (2.51) ^b^	64.60	67.65	72.30
2	FUS	External (A)	62.08 (0.92) ^c^	60.58	62.29	63.08
2	FUS	Internal (B)	53.14 (2.31) ^d^	50.10	53.12	57.00
3	XTF	External (A)	87.00 (1.97) ^e^	84.40	87.20	90.50
3	XTF	Internal (B)	82.80 (2.28) ^a^	78.90	83.00	86.20
4	GRA	External (A)	71.50 (1.70) ^f^	68.30	72.15	73.44
4	GRA	Internal (B)	65.60 (1.28) ^b^	63.80	65.70	67.20

SD: standard deviation; Min: minimum value; Mdn: median; Max: maximum value. Superscript letters (a, b, c, d, e and f) have been used to indicate statistical results: different letters indicate the presence of significant differences in micro-hardness among the groups (significance was set at *P* < 0.05).

**Table 3:** Mean percentage micro-hardness loss of the internal side of each tested material, compared to the external side.

Group	Material Code	Mean Percentage Loss (SD) %
1	VIS	−18.14 (0.02) ^a^
2	FUS	−14.40 (0.04) ^b^
3	XTF	−4.79 (0.03) ^c^
4	GRA	−8.22 (0.02) ^d^

SD: standard deviation. Superscript letters (a, b, c and d) have been used to indicate statistical results: different letters among the groups indicate significant difference in mean percentage loss among the groups (significance was set at *P* < 0.05).

**Table 4:** Hardness ratio of each tested material. SD: standard deviation.

Group	Material Code	Hardness Ratio (SD)
1	VIS	0.82 (0.02)
2	FUS	0.86 (0.04)
3	XTF	0.95 (0.03)
4	GRA	0.92 (0.02)

**Table 5:** Descriptive statistics of VHN for each tested material, considering two different acid storages compared to a group control.

Subgroups	Material Code	Storage	Mean (SD)	Min	Mdn	Max
1A	VIS	Control	82.82 (1.62) ^a^	80.10	83.21	85.10
1B	VIS	1-day acid drink	76.76 (1.75) ^b^	73.40	77.08	78.77
1C	VIS	1-week acid drink	71.70 (1.68) ^c^	69.60	71.65	74.70
2A	FUS	Control	62.08 (0.92) ^d^	60.58	62.29	63.08
2B	FUS	1-day acid drink	57.22 (1.03) ^e^	55.72	57.65	58.40
2C	FUS	1-week acid drink	53.80 (0.92) ^f^	52.30	53.90	55.00
3A	XTF	Control	87.00 (1.97) ^g^	84.40	87.20	90.50
3B	XTF	1-day acid drink	84.06 (2.02) ^a^	80.20	84.43	86.41
3C	XTF	1-week acid drink	79.04 (2.01) ^b^	76.40	79.57	81.60
4A	GRA	Control	71.50 (1.70) ^c^	68.30	72.15	73.44
4B	GRA	1-day acid drink	67.90 (1.35) ^h^	65.80	68.20	69.36
4C	GRA	1-week acid drink	62.96 (1.26) ^d^	61.30	63.08	64.90

SD: standard deviation; Min: minimum value; Mdn: median; Max: maximum value. Superscript letters (a, b, c, d, e and f) have been used to indicate statistical results: different letters indicate the presence of significant differences in micro-hardness among the groups (significance was set at *P* < 0.05).

**Table 6:** Mean percentage micro-hardness loss for the tested materials between different immersion protocols. T0: controls; T1: 1-day storage; T2: 7-day storage.

Group	Material Code	T0–T1 (%)	T1–T2 (%)	T0–T2 (%)
1	VIS	−7.30 (2.05) ^a^	−6.58 (1.85) ^a^	−13.42 (1.72) ^b^
2	FUS	−7.83 (0.57) ^a^	−5.97 (0.72) ^a^	−13.34 (0.50) ^b^
3	XTF	−3.36 (2.07) ^c^	−5.92 (3.55) ^a,c^	−9.12 (2.68) ^a,d^
4	GRA	−5.02 (1.27) ^a^	−7.27 (0.99) ^a^	−11.92 (1.77) ^b,d^

Superscript letters (a, b, c, and d) have been used to indicate statistical results: different letters indicate the presence of significant differences in mean percentage micro-hardness loss among the groups (significance was set at *P* < 0.05).

**Table 7:** Linear regression model of the variable Vickers hardness over time.

Coefficient	Estimate	Std. Error	t Value	Pr(>|t|)	Confidence	Intervals
Intercept	74.35	1.21	61.41	<0.0001	71.97	76.72
Time	−0.04597	0.01	−3.72	<0.0001	−0.07	−0.02
